# Caregivers’ expectations of their non-verbal autistic children in rural KwaZulu-Natal

**DOI:** 10.4102/sajcd.v71i1.1049

**Published:** 2024-09-27

**Authors:** Fatima Haffejee, Jennifer A.H. Pahl, Saira B. Karrim

**Affiliations:** 1 Discipline of Speech-Language Pathology, School of Health Sciences, University of KwaZulu-Natal, Durban, South Africa

**Keywords:** autism spectrum disorder, parents, expectations, caregivers, communication, speech-language therapy, rural, education

## Abstract

**Background:**

Caregiver expectations have been shown to impact child outcomes. There is limited research regarding caregivers of non-verbal autistic children in rural South Africa. Autistic individuals form part of a larger environment, which they influence and which impacts them. Caregivers form part of this environment.

**Objectives:**

This study aims to explore caregivers’ expectations of communication, education, social implications and intervention for their non-verbal autistic child in rural KwaZulu-Natal (KZN).

**Method:**

Bronfenbrenner’s ecological and bioecological systems theory framed the study and allowed the child’s interaction with their environment to be understood through the use of a qualitative study design via interviews. Eleven caregivers (pilot study: *n* = 1 and main study: *n* = 10) of non-verbal autistic children were selected and interviewed. Data were analysed thematically.

**Results:**

Caregivers had varied expectations. Grandparents were often the primary caregivers (microsystem). Relationships within the mesosystem (caregiver and therapist) and caregiver’s understanding affected their feelings and expectations that changed over time (chronosystem). Education was the predominant expectation. The study highlighted limited resources (schools) within the exosystem. Caregivers reported both support and judgement from their communities.

**Conclusion:**

There is a need for public awareness, caregiver counselling and autism support groups in rural KZN and more specialised education options in order to improve caregivers’ expectations.

**Contribution:**

This study contributes to the limited literature in the field of autism in South Africa, more specifically the rural context and communication disorders.

## Introduction

Autism spectrum disorder (ASD) is defined as a ‘pervasive neurodevelopmental disorder characterised by impairments in social communication and restricted, repetitive patterns of behaviour, interests or activities’ (American Psychiatric Association [APA], 2013, p. 18). Individuals with ASD vary in their communication abilities: some may present with superior language, while others present with significant impairment, for example, being non-verbal (APA, 2013; ASHA, 2019). Literature suggests that 25% – 40% of autistic individuals do not acquire verbal language (Anderson et al., 2007; CDC, 2019). Non-verbal (uses less than 10 words) (Springer et al., 2013) autistic children have reportedly poorer outcomes than their verbal counterparts (Patten et al., 2013). Upon receiving the diagnosis of ASD, caregivers often feel a loss of expectations that they had for a typically developing child (Byrne et al., 2018; Pottas & Pedro, 2016). Caregiver expectations are often related to communication, education, social participation, employment, therapy and the reactions of society.

There has been an increase in research specifically regarding caregivers’ experiences with ASD in South Africa (Mazibuko et al., 2020; Pillay et al., 2024; Van Niekerk et al., 2023). During the researcher’s community service year (in a rural hospital), it was observed that caregivers of non-verbal autistic children had been provided with very little information to aid in understanding autism, and there was no way of identifying the expectations of caregivers unless they were asked. No research regarding caregivers’ expectations of their non-verbal autistic children could be found internationally or locally at the time of this study being conducted. This study contributes to research by exploring the rural context in KwaZulu-Natal (KZN). The information from the study will enable speech-language therapists (SLTs) in South Africa, more specifically in rural KZN, to gain insight into what caregivers expect of their non-verbal autistic children. Awareness of the expectations of caregivers will guide health care professionals (HCPs) in educational counselling where these expectations can be managed.

### Aim and objectives

The aim of this study was to explore caregivers’ expectations for their non-verbal child with ASD in rural KZN. The objectives were to: (1) explore caregivers’ expectations regarding communication for their non-verbal autistic child, (2) explore caregivers’ expectations regarding the education of their non-verbal autistic child, (3) explore caregivers’ views regarding the social implications of having a non-verbal autistic child and (4) explore caregivers’ expectations regarding speech-language therapy intervention.

## Research methods and design

### Theoretical framework

A hybrid model of the ecological systems theory and the bioecological systems theory (Bronfenbrenner, 1979; Bronfenbrenner & Morris, 2006) was utilised as a framework that guided the study. The core components of the model are personal factors and processing factors. Personal factors like the autistic individual’s diagnosis and its impact on development and the interactions with their processing factors (microsystem, mesosystem, exosystem and chronosystem) were explored. The model was used to critically discuss caregivers’ expectations and gain perspective on how these expectations affect the autistic individual’s environment.

### Study design

A descriptive, qualitative, phenomenological case study design was utilised with interviews being conducted. Participants met the criteria of being a primary caregiver of a non-verbal autistic child between the ages of 5 and 18 years, residing in rural KZN (outside of a metropolitan), who have accessed or are accessing speech therapy for their autistic child. The author acknowledges that there is no standard definition of rural in South Africa (Gaede & Versteeg, 2011); however, rurality is more than geographical, and it also refers to ‘the structure, state and quality of life of people living in sparsely settled places away from the direct influence of large cities and towns’ (Duncan et al., 2011, p. 30).

### Study population and sampling strategy

Participants were recruited and purposively selected via public hospitals in rural areas in KZN. Twelve potential participants from five districts in KZN (Zululand, Ugu, UmGungundlovu, Amajuba and iLembe) responded and met the inclusion criteria. One participant was included in the pilot study. Table 1 illustrates the description of participants.

**TABLE 1:** Description of participants.

Participant code	Code for child with ASD	Age (years)	Gender	Race	Employment status	Language of interview	Relationship to child with ASD	Age of child with ASD (years)	Schooling	District of KZN
CGP	CWAP	76	Female	Black	Pensioner	isiZulu	Grandmother	7	Mainstream	Zululand
CG1	CWA1	31	Female	Black	Employed	English	Mother	7	Special Education	iLembe
CG2	CWA2	44	Female	Black	Employed	English	Mother	5	Not enrolled	Ugu
CG3	CWA3	30	Female	Black	Unemployed	isiZulu	Mother	5	Not enrolled	iLembe
CG4	CWA4	60	Female	Coloured	Employed	English	Grandmother	5	Mainstream	Amajuba
CG5	CWA5	41	Female	Black	Unemployed	isiZulu	Mother	9	Not enrolled	Amajuba
CG6	CWA6	57	Female	Black	Unemployed	isiZulu	Grandmother	5	Mainstream	Amajuba
CG7	-	33	Male	Black	Unemployed	-	Uncle	-	-	-
CG8	CWA8	43	Female	Black	Unemployed	English	Mother	7	Not enrolled	Ugu
CG9	CWA9	57	Female	Black	Part-time employment	isiZulu	Grandmother	7	Not enrolled	Ugu
CG10	CWA10	23	Female	Black	Student	English	Mother	5	Mainstream	umGungundlovu
CG11	CWA11	67	Female	Black	Pensioner	isiZulu	Grandmother	5	Enrolled	umGungundlovu

ASD, Autism spectrum disorder; KZN, KwaZulu-Natal; CWAP, child with autism pilot; CWA, child with autism; CGP, caregiver pilot; CG, caregiver.

### Data analysis

The interviews were transcribed. The isiZulu interviews were transcribed by the interpreter. The isiZulu transcript was sent to another interpreter to translate back into English to ensure that the responses were consistent, the English transcripts were cross-checked against recordings for accuracy and sense-making by the researcher. The data were organised using a table of sources. A preliminary, exploratory, inductive analysis was conducted (Creswell, 2012). Thereafter, the data were analysed by colour coding the information to narrow the data into themes and form descriptions (Creswell, 2012). The final step of data analysis, the interpretation, was conducted by comparing the data to the literature and the hybrid theory that framed the study (Bronfenbrenner, 1979; Bronfenbrenner & Morris, 2006; Creswell, 2012).

### Trustworthiness

The issues of dependability, credibility, transferability and confirmability were considered in order to ensure the trustworthiness of the data. Credibility was ensured by using two high-quality recorders during interviews and member checking by allowing the participants to view the transcripts for approval after the interviews (Modaff & Modaff, 2000). Transferability was ensured by the use of thick description, which refers to describing not just the experiences and behaviours of the caregivers of the autistic individual but their contexts as well (Tracy, 2010). Dependability and confirmability were ensured by the use of an audit trail (Tracy, 2010), which is the documenting and describing of the steps taken from the beginning of the research to the end and keeping records (Lincoln & Guba, 1985).

### Ethical considerations

The data collection procedure included the researcher obtaining ethical clearance from the University of KwaZulu-Natal (UKZN) Biomedical Research Ethics Committee (BREC) with reference number BREC/00000096/2019. A letter of support was granted by the programme manager at the KZN Department of Health Research. Thereafter, permission was granted by the KZN Department of Health to access public hospitals in areas that are classified as rural, that is, areas out of a metro (Gaede & Versteeg, 2011). This was conducted via an e-mail letter and a telephonic follow-up. Potential participants were identified by the resident speech-language therapists at the hospitals that the caregivers access for their autistic children, and information documents were sent to potential participants; potential participants with reduced literacy were provided information regarding the study telephonically. Participants were only included once the consent was provided. Semi-structured, face-to-face interviews were conducted in either English (five interviews) or isiZulu (six interviews), depending on the preference of the participant. An interpreter who was trained by the researcher was involved when the participant requested the interview to be conducted in isiZulu. The interview was audiorecorded on two high-quality Dictaphones (Panasonic IC recorder RR-XS350). The interview schedule followed the themes of the core literature and consisted of 9 sections with 35 open questions and 16 probes. The pilot study was done to trial the procedure, data collection instruments and analysis methods, after which changes were made. Therefore, the pilot participant information and data were not included in the main study.

The results of the pilot study indicated a need to make minor adaptations to the consent and indemnity forms. These changes were made to accommodate [reduced] literacy levels. Additional probes were included in the interview schedule, and changes to the isiZulu translation methods were implemented. Potential participants were identified by the speech-language therapists at the rural hospitals. The study was explained explicitly telephonically to participants, and an information document was provided. Proxy consent was applied for participants with reduced literacy. The researcher or interpreter verbally explained the study in detail to the participants before they could sign. The risk of psychological distress such as feelings of anxiety and depression related to discussing this sensitive topic was considered. Participants were given the opportunity to stop the interview at any point and be provided with a list of public and private psychologists nearest to them. Each interview lasted approximately 1 h.

## Results and discussion

**FIGURE 1:**
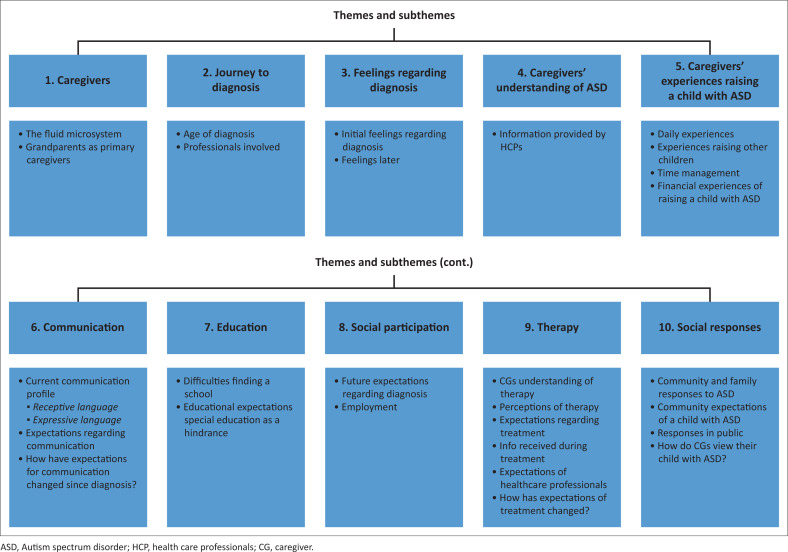
Themes and subthemes.

Ten primary themes and subthemes emerged during the data analysis (see Figure 1). The 10 themes included caregivers’ journey to and feelings regarding diagnosis, understanding of ASD, experiences raising an autistic child and expectations regarding communication, education, social participation, employment, therapy and, lastly, societal responses.

### Caregivers – the fluid microsystem

‘Well actually I am staying inside the __ so it’s a single quarters, she stays with my mum and my sister but most of the time she is with my mum because she is always at home, my sister is always working … my mum has the responsibility.’ (CG2)

The autistic children’s microsystem was fluid, with caregivers often not being biological parents and changing frequently. Many young, black South African rural residents leave to seek employment in urban areas leaving other family members, often grandparents to care for their children (Schatz & Ogunmefun, 2007). It results in HCPs liaising with multiple individuals, which could affect the consistency of care and create challenges when providing counselling.

### Journey to diagnosis

‘Honestly the doctor, didn’t really communicate anything with me, he write it down … I read the diagnosis down on the card. He just told me that his milestones are delayed and when I read the card thoroughly, I see that there is ASD.’ (CG10)

Obtaining a diagnosis of ASD is a complicated and lengthy process (Nadel & Poss, 2007). Children in this study generally received a diagnosis early (between 18 months old and 4 years old) in comparison to literature (after 3 years old) (Mitchell & Holdt, 2014). Three caregivers were unsure of the exact age of diagnosis, which could be attributed to the fluid microsystem.

A correlation between early diagnosis and private health care was identified as CWA1 was diagnosed at 18 months old. Two caregivers reported not being told the diagnosis, three were informed by the SLT, one was informed that the ‘blood test’ was negative but the child presented with characteristics of autism and one participant (CG10) discovered the ASD diagnosis from reading the patient hospital card. The role of traditional media in rural KZN was highlighted when CG6’s husband read about the characteristics of ASD in a local newspaper and sought help. Two caregivers reported not receiving a diagnosis. CG11 reported that she did not understand the term ASD when counselled as it was in English. Because of the influence of colonisation and apartheid on research practices, there are limited resources in indigenous languages (Pillay & Kathard, 2015).

### Feelings regarding the diagnosis

‘Kwaba buhlungu kakhulu ngoba phela uma ubuza ukuthi lento izolapheka yini, bathi cha. [*It was very painful because you ask if it’s curable and they say no*].’ (CG11)

Caregivers often feel a strong sense of emotion when hearing that their child is autistic, and many of these feelings have to do with losing the image of the ‘perfect child’ (Gentles et al., 2020). Caregivers felt upset, overwhelmed, guilty and stressed upon receiving the diagnosis. CG2 reported feelings such as intense loss. The participant mentioned that she would have preferred her child to have died, while CG9 resorted to excessive use of alcohol. CG8 felt denial and did not follow up on the referrals. She obtained a diagnosis 3 years later, which led to no access to beneficial early intervention services. Denial is a common reaction that caregivers of autistic children experience immediately after diagnosis (Gentles et al., 2020). CG4 reported a feeling of acceptance as she knew that CWA4 had difficulties. Research suggests that caregivers who suspect a developmental delay have more positive reactions to an ASD diagnosis than caregivers who do not suspect that their children have difficulties (Nissembaum et al., 2002):

‘When the psychologist told me that, at the back of my mind, I knew that he had a problem. I already knew and my expectations is that he won’t be like a normal child.’ (CG4)

Despite initial negative reactions, most caregivers’ views changed. Speaking to other caregivers and support groups was found beneficial, which has been supported by research (Gerber, 2014). Access to some support groups was limited to families with children at a local school. Three caregivers (CG3, CG6 and CG8) still feel sadness when they see younger children surpass their autistic children, particularly in language development. This is consistent with research by Bravo-Benítez et al. (2019), which stated that the feelings of grief regarding raising an autistic child are cyclic with periods of happiness and acceptance and feelings of anguish and sadness thereafter.

### Caregivers’ understanding of autism spectrum disorder

‘According to the psychologist, she told me it’s the condition of the mind. I think it something with the brain, just brain damage.’ (CG5)

Most caregivers understood ASD based on information presented to them by their HCPs. Some caregivers had done their own research. Two caregivers mentioned it being a spectrum, and many caregivers described brain abnormalities, which is possibly how HCPs had described ASD to them. Language of communication with HCPs may have also influenced their understanding, as those who were not English speakers reported having a poorer understanding of ASD and its implications in comparison to English speakers; this is supported by Levin (2014) who stated that in South Africa, consultations are often done in a patient’s second or third language:

‘Unegqondo okusengathi incane futhi akakwazi ukukhuluma. [*His brain seems smaller and he can’t talk*].’ (CG3)

### Experiences raising autistic children

‘I’d say that he is quite difficult to raise, because basically I am his mind, I have to think everything for him, I have to think no, he is hungry now, he should go to the toilet now.’ (CG10)

Nine caregivers described raising a child with ASD as challenging and stressful. Toilet training and self-injurious behaviours made leaving their children in the care of others difficult and were the primary concern, possibly as this affected school placement. CG10 was the only participant who was concerned about communication for the future. Three caregivers were worried for the child’s future when they pass away, and contrastingly, one caregiver described raising her grandson as easier than raising her children:

‘[*Raising CWA4 is*] Wonderful, I would never say even with my own children I had sleepless nights, with this child I never had a sleepless night.’ (CG4)

Family unity and the role of siblings were highlighted. Financial difficulties forced CG9 to make CWA9’s health care appointments further apart so that she could afford transport. Five out of the eleven participants were unemployed, only one (CG11) was eligible for an old age pension, three were employed full-time, two worked part-time and three children received disability grants. Autism is regarded as the most expensive disability (Byford et al., 2015).

Finances were further burdened by purchasing sensory toys and picky eating resulting in buying specific foods and accessing services:

‘His diet isn’t stable, so sometimes it gets difficult financially because we have to buy different foods.’ (CG7)

### Communication

‘I think he prefers not to follow instructions because he can hear clearly.’ (CG10)

Five caregivers reported that their children had good receptive language and ‘understand everything’, while four caregivers reported that their children presented with inconsistent understanding. One caregiver mentioned that their child responds only when they want to. The rolling blackout in South Africa was mentioned as a challenge as one child did not understand this and becomes frustrated with the disruption of routine. Expressive language was described as verbal language (CG1, CG4 and CG10), consistent pointing (CG1) and inconsistent pointing (CG3, CG8 and CG9). Seven caregivers reported that their children would often fetch desired items themselves or use hand-leading. Caregivers saw no need for requesting if children were independent in their routines. Caregivers’ expectations of communication varied and had changed since diagnosis. Some caregivers based their expectations on the progress that their child had displayed thus far and not on intellectual functioning. Expectations included verbal communication, functional communication (CG1) and competent communication (CG8). Caregivers felt that the child would improve based on the progress since diagnosis, while others expected improvements despite seeing no changes. A few based their expectations on what they considered attainable, e.g., following simple instructions. It is a common misconception held by caregivers that AAC hinders their child’s speech and language development (Romski et al., 2010; White et al., 2021). This sentiment was reported by CG8 who felt that ‘signing’ would not be beneficial:

‘He talks one word so each time he says that word I get happy so we are going somewhere, at home we speak. We don’t use signs. I don’t want anyone to use signs with my child because I feel like if they use signs he will not grow and develop that is what I feel.’ (CG8)

### Education

‘Kunzima ukuthola isikole, ngoba phela bavele bakutshele ukuthi uzongena kuhlu lokulinda, uyoze aye esikoleni emva kweminyaka ewu-4 noma-5 elindile. [*Finding a school is difficult because they just tell us that he’ll be on their waiting list, he’ll only go to school after 4 or 5 years of waiting*].’ (CG11)

Five of the ten children with ASD currently attend school, one (CWA1) at a special needs school and four at mainstream pre-schools (CWA4, CWA6, CWA9 and CWA10). Caregivers reported poor communication from schools, being on a waiting list for up to 3 years, finances (transport costs) and criteria for acceptance, such as children having to be toilet trained as barriers to special needs education:

‘I heard that the schools are very expensive. The government schools- I have tried- but they said no, she is wearing nappy, they cannot take her.’ (CG2)

While there are government policies (the macrosystem) in place for inclusive education [e.g. White Paper 6 (Department of Education, 2001)], the resources available, e.g., trained teachers and appropriate information, are insufficient (exosystem) and possibly even less available in rural areas (Donohue & Bornman, 2014). Parents opted for schools close to home as child safety and sexual abuse of people with disabilities were factors to consider (Mdikana et al., 2018). Some caregivers expected a slight improvement in education (CG2, CG3, CG6, CG8, CG9, CG10 and CG11), while others expected it to lead to tertiary education and careers (CG1, CG4 and CG5). Literacy was a desired outcome but not an expectation. Some caregivers thought that being surrounded by other children with disabilities may have a negative impact:

‘Sengibonile ukuthi ngoba kugcwele izingane ezikhubazekile uzogcina naye egqilazeka emqondweni. [*I’ve noticed that there are a lot of children with disabilities so that will exhaust his mind*].’ (CG3)

### Social participation and employment

‘[*laughs*] CWA8 doesn’t have friends, Haibo, CWA8 doesn’t have any friends, CWA8 doesn’t play with other children. At my mother’s place there are children, my brother’s children, young children, but he prefers to play with the goats [*laughs*].’ (CG8)

Persistent deficits in social participation and social development form part of the core characteristics of ASD (APA, 2013). Most caregivers reported that their children do not have friends as they did not attend school and displayed violent behaviour towards other children. There were no reports of social exclusion or bullying, as found by Rowley et al. (2012). This could be attributed to the close-knit communities in rural living. Caregivers expected social participation to improve with schooling, while others hoped for their children to have their own family and friends. Caregivers placed their expectations for employment on education. None of the caregivers mentioned communication or social deficits as a barrier to employment. Caregivers raised concerns that siblings could be overburdened with caregiving if autistic children became unemployed adults.

### Therapy

‘Bayazama, ayikho into engingayisho. Bayazama ukumusiza kodwa kushuthi sekukuyena [CWA6] manje. [*They try, what can I say … they try but it is up to him* {*CWA6*} *now*].’ (CG3)

All caregivers reported that their children currently receive speech-language and occupational therapy at their regional public hospitals. Most participants understood therapy as a means to assist development. Three caregivers were unclear regarding the purpose of therapy and had difficulties differentiating between speech and occupational therapy. These caregivers also felt that therapy would not help their child, and they explained that this was because of them not understanding the purpose of therapy. This is consistent with a study done by Wetherston et al. (2017) in KZN, which reported that 78.2% of its participants were not informed about different types of treatment before beginning therapy. CG2 worked away from home and depended on her mother for feedback. Three caregivers reported changes in expectations over time as they went from seeing therapy as an immediate solution to it being a process after witnessing their child’s progress. Despite all autistic children being non-verbal, only two caregivers had expectations for communication, one to help ‘loosen the tongue’ and the other as a ‘cure’. Some caregivers had more specific expectations, such as assistance with picky eating (CG3) or medication (CG7). Caregivers reported receiving counselling regarding ASD and how to cope (3) or receiving inadequate information with no resources or counselling provided in their first language. It was found that expectations were not linked to *if* the caregivers receive information from HCPs but rather *how* the information is shared.

### Societal responses

‘Don’t even go there. Some they will say, she needs “emasiko” or the rituals.’ (CG2)

Participants lived in newly developed settlements in a rural area with no electricity or running water (CG5) to a more developed area with amenities (CG1). Most caregivers reported that their children were the first autistic person in the community. Negative views included community members believing that behaviours were related to bad parenting, the child being ‘mad’ or needing traditional healing (CG1, CG2 and CG10):

‘You know raising a child with autism sometimes appears to be naughty but he is not naughty, he is just dealing with autism.’ (CG10)

Most children with ASD and their caregivers enjoyed close relationships and interacted daily with members of their communities. Some caregivers experienced very supportive communities (CG5 and CG6), who enjoy spending time with the children and spoiling them (CG4 and CG9). Some caregivers felt upset with their child’s delayed milestones and behavioural challenges, with one grandmother reportedly physically abusing the child. One mother did not visit or contact her son, which CG9 attributed to his diagnosis. All caregivers reported that they loved their children in any case and wanted the best for them.

### Limitations

There were no participants in the 8–18 years of age range.

### Implications

Clinical implications include the need for in-depth counselling for caregivers and teachers regarding long- and short-term outcomes as well as therapy in order to improve caregiver expectations. Resources for assessment and intervention need to be created and made available to HCPs in different languages. A checklist of appropriate referrals for HCPs should be developed. Research implications include exploring the expectations of caregivers in other provinces and how teachers in mainstream schools in rural communities understand ASD and the role of support groups.

## Conclusion

**FIGURE 2:**
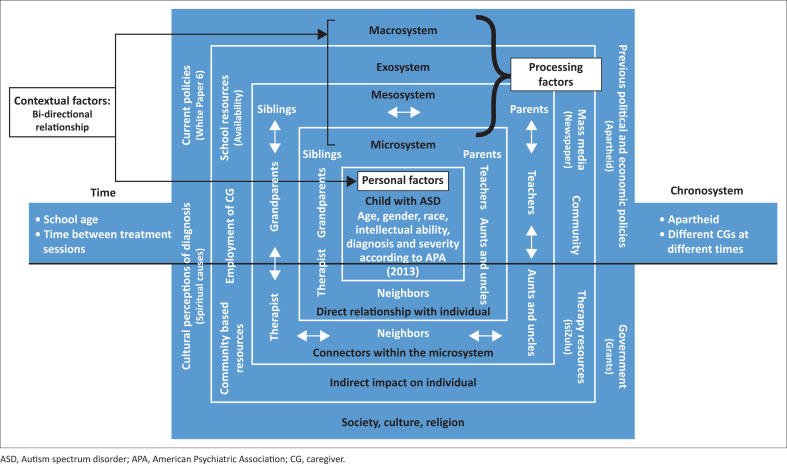
The hybrid theory (Bronfenbrenner, 1979; Bronfenbrenner & Morris, 2006) in relation to this study. This figure illustrates the results of the study (in white font) framed by the components of the hybrid theory (in black font).

The study highlighted the expectations of caregivers for their non-verbal autistic children in rural KZN. Many factors shaped caregivers’ expectations, as seen in Figure 2. These include personal factors such as the child’s age and diagnosis and processing factors such as the fluid microsystem, the relationship between HCPs and caregivers (mesosystem), the lack of educational options and resources available to HCPs (exosystem), the expectations of the community (macrosystem) and previous governmental policies (chronosystem). Education was a dominant theme running throughout the study taking precedence over communication and social expectations. There is a need to provide counselling for caregivers of non-verbal children with ASD as well as resources to HCPs to assist with counselling.
